# Patterns of Early Rejection in Renal Retransplantation: A Single-Center Experience

**DOI:** 10.1155/2016/2697860

**Published:** 2016-12-08

**Authors:** Lan Zhu, Cheng Fu, Kailin Lin, Zhiqiang Wang, Hui Guo, Song Chen, Zhengbin Lin, Zhishui Chen, Gang Chen

**Affiliations:** ^1^Institute of Organ Transplantation, Tongji Hospital, Huazhong University of Science and Technology, Wuhan, China; ^2^Key Laboratory of Organ Transplantation, Ministry of Education, Wuhan, China; ^3^Key Laboratory of Organ Transplantation, Ministry of Public Health, Wuhan, China

## Abstract

It has been reported that kidney retransplant patients had high rates of early acute rejection due to previous sensitization. In addition to the acute antibody-mediated rejection (ABMR) that has received widespread attention, the early acute T-cell-mediated rejection (TCMR) may be another important issue in renal retransplantation. In the current single-center retrospective study, we included 33 retransplant patients and 90 first transplant patients with similar protocols of induction and maintenance therapy. Analysis focused particularly on the incidence and patterns of early acute rejection episodes, as well as one-year graft and patient survival. Excellent short-term clinical outcomes were obtained in both groups, with one-year graft and patient survival rates of 93.9%/100% in the retransplant group and 92.2%/95.6% in the first transplant group. Impressively, with our strict immunological selection and desensitization criteria, the retransplant patients had a very low incidence of early acute ABMR (6.1%), which was similar to that in the first transplant patients (4.4%). However, a much higher rate of early acute TCMR was observed in the retransplant group than in the first transplant group (30.3% versus 5.6%, *P* < 0.001). Acute TCMR that develops early after retransplantation should be monitored in order to obtain better transplant outcomes.

## 1. Introduction

Renal transplantation is regarded as the optimal treatment for patients with end-stage renal disease. However, as long-term graft survival is still limited, most transplant patients will face graft loss after 9-10 years [1]. These patients are generally more fragile and in considerable need of new grafts, in comparison to naïve patients waiting for their first renal transplantation.

It has been reported that the best approach to treat most patients suffering from chronic renal allograft failure is to perform a kidney retransplant, in hopes of avoiding the high risk of morbidity and mortality with a return to dialysis [2]. These patients, however, are commonly human leukocyte antigens- (HLA-) sensitized because of exposure to previous allograft(s); thus there is a lower chance of their receiving a retransplant. Retransplantation accounts for 13–15% of the annual transplants performed in USA and only approximately 5% of those performed in Europe [3]. Therefore, every retransplant case needs to be evaluated and managed very carefully.

Renal retransplant patients had high rates of acute rejection, from 33% to 69%, as reported in previous studies [4–6]. About two-thirds of these rejections were verified as antibody-mediated rejection (ABMR), comprising the primary cause of early graft loss. Thus, it is well recognized that the risk of ABMR in retransplantation increases markedly and needs to be prevented as much as possible. In contrast, the risk of T-cell mediated rejection (TCMR) in retransplantation is less of a concern. Compared to first transplant patients, it is unclear whether the incidence of acute TCMR would significantly increase in retransplant patients without early ABMR. In other words, if de novo donor-specific antibody (DSA) and its mediated ABMR could be prevented successfully in retransplantation, would TCMR be brought to the forefront as an important issue?

Here, we report on the early transplant outcomes of 33 second, third, and fourth kidney transplants performed at our hospital within the last 3 years. Analysis focused particularly on the incidence and patterns of the early acute rejection episodes, as well as one-year graft and patient survival.

## 2. Patients and Methods

### 2.1. Study Population

Between January 2013 and December 2015, a total of 703 kidney transplants were performed at Tongji Hospital, including 521 transplants from deceased donors (donation after brain death or cardiac death) and 182 from living-related donors. Of these, 662 (94%) were first transplantations and 41 (6%) were retransplantations.

In the current retrospective study, for the retransplant group, we included 33 adult patients, who received a second, third, or fourth renal allograft with Thymoglobulin induction therapy and Tacrolimus-based maintenance therapy. The exclusion criteria were as the following: (1) pediatric recipients; (2) renal allografts from pediatric donors; (3) patients who received no induction therapy or received induction therapy other than Thymoglobulin; (4) patients who received a multiorgan transplant. For the control group, we selected 90 patients who received a first renal allograft during the same period and fulfilled the same inclusion and exclusion criteria. This study was performed after approval by the ethics committee at Tongji Hospital, Tongji Medical School, Huazhong University of Science and Technology.

### 2.2. Data Collection

Data on transplantations and hospital stays, as well as follow-up data, were collected from hospital records. Baseline characteristics, such as recipient age and gender, donor type (deceased or living), number of previous transplants, cold ischemia time, number of HLA mismatches, pretransplant panel reactive antibody (PRA) percentages divided into groups (0–10%, >10%–50%, and ≥50%), and preformed DSA, were collected and analyzed. In addition, early clinical outcomes, including the generation of de novo DSA, rate of delayed graft function (DGF), the frequency and type of acute allograft rejection (cellular or antibody-mediated rejection), and one-year graft and patient survival, were analyzed. DGF was defined as the need for more than 1 dialysis during the first week after transplant. HLA class I and II antibody screenings were performed using FlowPRA® (One Lambda, Inc., Canoga Park, CA), and the specificity determination was measured by Luminex using LABScreen® single antigen beads (One Lambda, Inc., Canoga Park, CA).

### 2.3. Immunosuppression

All patients received induction therapy with Thymoglobulin and maintenance immunosuppression with Tacrolimus, mycophenolate mofetil, and steroids. Because of differences in immunological risk, the dosage and duration of the Thymoglobulin administration were slightly different for the retransplant group, compared to the control group. The initial administration of Thymoglobulin was finished intraoperatively, before the graft reperfusion, at a dose of 50 mg in the retransplant group and a dose of 25 mg in the control group. Then, it was used daily by the retransplant group from day 1 to day 4, reaching a total dosage of 125–150 mg. In the control group, Thymoglobulin was given from day 0 to day 2 at a daily dose of 25 mg. The peripheral blood lymphocyte counts were frequently monitored within the first 2 weeks after renal transplantation. Methylprednisolone was given intravenously from day 0 to day 2 (500 mg/d), followed by oral doses of prednisone at 50 mg/d, which was then tapered every other day to a maintenance dose of 10 mg/d. Mycophenolate mofetil was administered at a dose of 1.5 g/d and was subsequently reduced to 1 g/d depending on the individual's white blood cell count. Tacrolimus was started at day 3, with a targeted trough level of 8–10 ng/ml initially and 6–8 ng/ml one month after transplantation.

### 2.4. Desensitization

**Figure 1:**
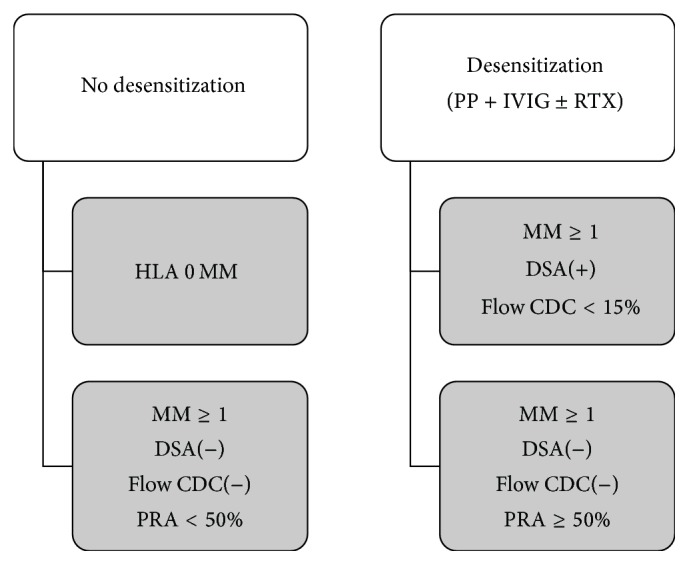
Indications of desensitization therapy for our retransplant patients. No desensitization was initiated if patients had HLA 0 MM, or HLA MM ≥ 1, but PRA < 50% with negative DSA and CDC. Desensitization was suggested if patients had (1) HLA MM ≥ 1, DSA positive, and negative or weakly positive flow CDC (<15%) or (2) HLA MM ≥ 1 and PRA ≥ 50% with negative DSA and CDC.

In our center, the decision of desensitization was mainly based on the laboratory testing results for pretransplant PRA, preformed DSA, HLA mismatch (MM), and flow complement-dependent cytotoxicity (CDC). As shown in Figure 1, sensitized patients were not required to be desensitized in the following two situations: (1) HLA 0 MM between donor and recipient and (2) HLA MM ≥ 1, PRA < 50% with negative DSA and flow CDC. In contrast, desensitization was suggested for sensitized patients in any of the following conditions: (1) HLA MM ≥ 1, DSA positive, and negative or weakly positive flow CDC (10–15%); (2) HLA MM ≥ 1 and PRA ≥ 50% with negative DSA and flow CDC. For a few highly sensitized patients with flow CDC ≥ 15%, the upcoming transplantation was then avoided due to the limitations of current desensitization therapy. The protocol we used to desensitize patients involved a combination of plasmapheresis (PP), intravenous immunoglobulin (IVIG), and/or rituximab (RTX). IVIG was used at a dose of 300–400 mg/kg each time and RTX was given once at a dose of 200 mg.

### 2.5. Diagnosis of Acute Rejection

In general, acute rejection was diagnosed using kidney biopsies upon Banff 2007 or 2013 classification. When a tissue analysis was not available, the clinical diagnosis was based on an otherwise unexplained elevation of serum creatinine levels, coupled with appropriate physical signs (including edema, oliguria, fever, or weight gain). All allograft biopsies were routinely stained for hematoxylin and eosin (HE) and immunohistochemistry for C4d, CD3, CD4, and CD8. The diagnosis of acute TCMR was based on the following criteria: IA, cases with significant interstitial infiltration (>25% of parenchyma affected, i2 or i3), and foci of moderate tubulitis (t2); IB, cases with significant interstitial infiltration (>25% of parenchyma affected, i2 or i3), and foci of severe tubulitis (t3); IIA, cases with mild-to-moderate intimal arteritis (v1); IIB, cases with severe intimal arteritis comprising >25% of the luminal area (v2); III, cases with “transmural” arteritis and/or arterial fibrinoid change and necrosis of medial smooth muscle cells with accompanying lymphocytic inflammation (v3) [7]. Acute ABMR was defined as a biopsy with or without C4d, evidence of acute renal injury and microvascular inflammation, in the presence of circulating DSA [8]. If an episode of ABMR occurred together with acute TCMR, it was also defined as ABMR. Early acute rejection referred to a rejection that developed < 90 days after transplantation.

### 2.6. Statistical Analysis

In our descriptive statistical analysis, results are expressed as numerical values and percentages for categorical variables and as mean values with standard deviation (SD) for continuous variables. The frequencies procedure was used to compare baseline characteristics between the two groups. Graft and patient survival was evaluated according to Kaplan-Meier survival statics. Statistical analysis was performed using SigmaStat software version 3.5.

## 3. Results

### 3.1. Patient Population

The patient cohort included 33 kidney retransplants and 90 first transplants. The retransplant group comprised 28 (84.9%) second, 4 (12.1%) third, and 1 (3%) fourth kidney transplants. Baseline and demographic characteristics are shown in Table 1. The mean recipient age and sex ratios were similar in both groups. The majority of patients received kidneys from deceased donors (72.7% in retransplant group and 100% in control group). No statistical difference was observed between the two groups in terms of cold ischemia time and the global HLA mismatch (3.2 ± 1.2 in the retransplant group versus 3.5 ± 1.2 in the control group). However, in relation to the pretransplant PRA levels, a much higher incidence of HLA presensitization was present in the retransplant recipients than in the first transplant recipients (57.6% versus 3.3%, *P* < 0.001). In particular, 11 (33.3%) retransplanted patients were highly presensitized, as evidenced by the high pretransplant PRA levels that were detected (class I or class II ≥ 50%). Based on our criteria, 7 sensitized patients, including 3 patients with positive preformed DSA, received desensitization therapy to decrease anti-HLA antibody levels and ensure a negative flow CDC before their retransplants. Early transplant outcomes were then evaluated on the basis of the frequency of DGF and acute rejection, as well as one-year graft and patient survival.

### 3.2. DGF

The incidence of DGF in both groups was not high, with 12.1% in the retransplant group and 17.8% in the control group (*P* > 0.05). All patients with DGF were able to achieve normal renal function within 2–4 weeks.

### 3.3. Acute ABMR

The rates of acute ABMR in both groups were similarly low, with 6.1% (2/33) in the retransplant group and 4.4% (4/90) in the control group (*P* > 0.05). In the retransplant group, two patients unexpectedly generated large amounts of de novo DSA in the first week after transplantation, resulting in severe acute ABMR of renal allografts and eventual graft loss. In the control group, 3 grafts developed mild or moderate acute ABMR, which was successfully reversed by treatment with PP + IVIG. Unfortunately, severe acute ABMR was also seen in the control group, leading to graft loss in 1 patient.

### 3.4. Acute TCMR

**Figure 2:**
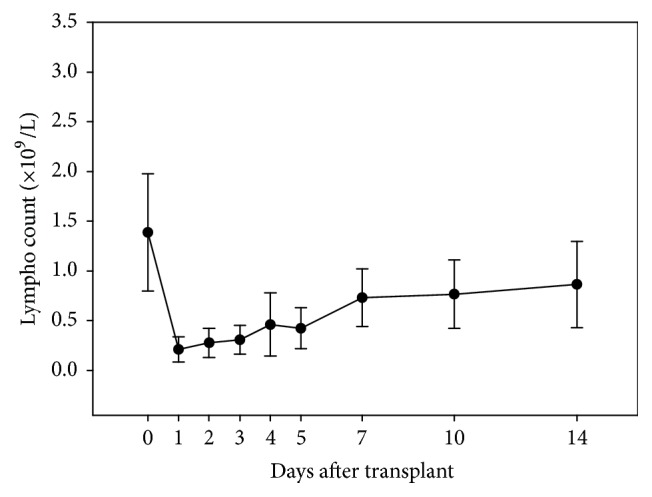
The average blood lymphocyte counts in retransplant patients before and after Thymoglobulin induction therapy. A satisfactory decline of peripheral blood lymphocyte count in the retransplant group was achieved with a 5-day period of Thymoglobulin induction therapy (total dosage: 125–150 mg).

In the control group, acute TCMR was only seen in 5.6% (5/90) of the patients with an induction therapy of low Thymoglobulin doses. However, even when a higher total dosage of Thymoglobulin was administered, resulting in a satisfactory decline in peripheral blood lymphocyte count (Figure 2), a much higher rate of TCMR was still observed in the retransplant group (30.3% versus 5.6% in the control group, *P* < 0.001). A total of 14 acute TCMR episodes were observed in 10 of 33 retransplant patients. Among them, 4 patients had 2 sequential episodes of early acute TCMR. The acute TCMR usually occurred around 2 weeks after the retransplantation and could be successfully reversed by either the high-dose steroid pulse therapy alone or its combination with Thymoglobulin.

**Figure 3:**
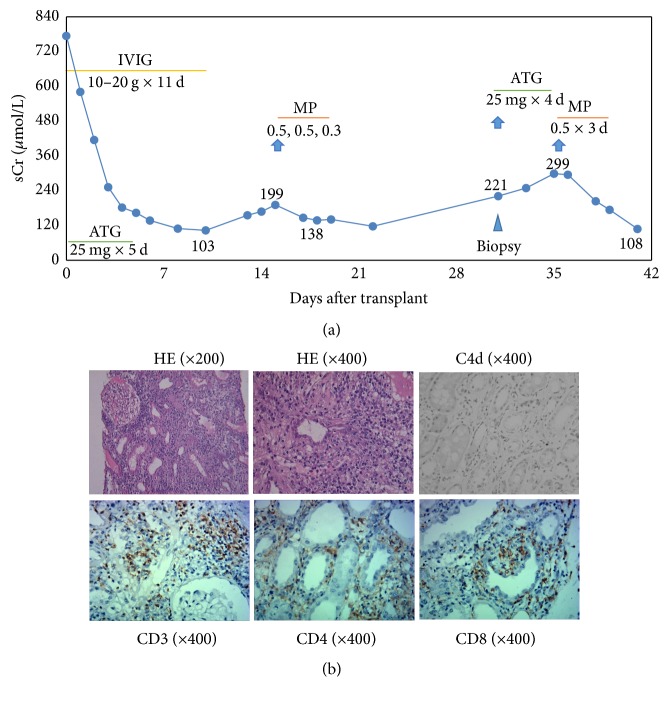
The clinical course of a second graft recipient who had early acute TCMR twice after transplantation. A 47-year-old male patient received his second kidney transplantation. (a) Early clinical course after retransplantation: renal graft gained immediate function with serum creatinine (sCr) levels decreasing rapidly and reaching normal levels at day 7. Increased sCr was observed at around day 14 and 3 doses of Methylprednisolone (MP, 500 mg/d) were then administered. After treatment, sCr levels decreased to 117 *µ*mol/L at day 23 and then were elevated again (221 *µ*mol/L) at day 31, and a biopsy was performed. sCr levels returned to normal after treatment with Thymoglobulin (25 mg/d) for 4 days and subsequent MP (500 mg/d) for 3 days. (b) Pathological results indicated acute TCMR, Banff 2007 grade IIA (i3, t2, v1, and g0), with extensive T-cell infiltration (positive staining for CD3, CD4, and CD8) and negative C4d staining.

A typical example of TCMR in the retransplant group is shown in Figure 3. The recipient was a 47-year-old man. His first renal allograft was from his sister (half HLA match), and the graft developed chronic failure after 10 years. He returned to dialysis without discontinuation of his immunosuppressive drugs. His preretransplant PRA levels were not significantly elevated (class I: 18% and class II: 2%). For his second transplant with HLA 3 MM, the flow CDC was negative. To prevent ABMR, IVIG was given at a daily dose of 10–20 g from day 0 to day 11. The second renal graft achieved immediate function with serum creatinine (sCr) levels decreasing rapidly and reaching normal levels at day 7. However, increased sCr levels were subsequently observed at around day 14 without detected DSA. Acute TCMR was the suspected clinical diagnosis, and thus 3 doses of Methylprednisolone (MP, 500 mg/d) were administered. After the treatment, the patient's sCr levels decreased to 117 *µ*mol/L at day 23. Surprisingly, at day 31 after transplantation, the patient once again had elevated sCr levels (221 *µ*mol/L), and a biopsy was performed (Figure 3(a)). Pathological results indicated acute TCMR, Banff 2007 grade IIA (i3, t2, v1, and g0), with extensive T-cell infiltration and negative C4d staining (Figure 3(b)). The patient fully recovered after treatment with Thymoglobulin (25 mg/d) for 4 days and subsequent MP doses (500 mg/d) for 3 days (Figure 3(a)).

Interestingly, almost all the episodes of TCMR were observed in lower presensitized retransplant patients with pretransplant PRA < 50% (Table 2). In contrast, only 1 of 10 highly sensitized patients (PRA ≥ 50%) developed acute TCMR. This patient did not receive desensitization due to 0 MM of HLA to his donor. Furthermore, none of the highly sensitized patients with desensitization therapy (*n* = 7) developed acute TCMR, indicating that desensitization may have some role in the prevention of early TCMR (Table 2).

### 3.5. One-Year Graft and Patient Survival

Both groups had high rates of graft and patient survival at the one-year mark (Table 1). In the retransplant group, graft and patient survival at 1 year were 93.9% and 100%, respectively. None of the retransplant patients lost their grafts due to early acute TCMR. Renal allograft failure was only seen in 2 retransplant patients, as a result of early severe acute ABMR. In the control group, graft and patient survival at 1 year were 92.2% and 95.6%, respectively. One female patient lost her graft in the early period after transplantation because of severe acute ABMR. Two patients lost their grafts due to renal artery rupture caused by donor-derived infections of* Candida albicans*. Additionally, the other 4 patients in the control group died with a functioning graft: 3 patients died from severe interstitial pneumonia which might be cytomegalovirus- (CMV-) relevant, while 1 patient died of cardiac arrest during surgery for renal graft rupture.

## 4. Discussion

Since the recipients of retransplantation are usually sensitized to certain mismatched HLA antigens because of exposure to previous allograft(s), they are at high risk for the development of acute ABMR. This process is mediated by either preformed or induced DSA that is produced as a result of an anamnestic response by memory B cells [9]. Consequently, considerable efforts have been made to prevent ABMR in kidney retransplantation, including a stringent immunological selection of donors, pretransplant desensitization therapy, and more potent induction therapy. As a result, second kidney transplants have been reported to have similar outcomes to first transplants [10]. Since the HLA presensitization may also result in the generation of alloantigen-specific memory T cells, which could mediate the so-called second-set rejection that is rather difficult to block or inhibit [11, 12], it is important to investigate the incidence and patterns of acute rejection in kidney retransplantation. In the current retrospective study, we included 33 retransplant patients and 90 first transplant patients with similar protocols of induction therapy and maintenance therapy. Excellent short-term clinical outcomes were obtained in both groups, as evidenced by high rates of one-year graft and patient survival and low incidences of early acute ABMR. Even so, a much higher rate of early acute TCMR was observed in the retransplant group than in the first transplant group, which has been rarely reported or emphasized in the literature.

It is well known that despite attempts devoted to improving outcomes in highly sensitized patients by using desensitization protocols, acute ABMR rates remain high, afflicting about 28%–40% of all cases [13, 14]. In our cohort, however, a much lower incidence of acute ABMR (6.1%) was observed in retransplant patients, leading to significantly improved short-term graft survival. This result is different from previous studies, which could be explained by our cautious selection of patients and donors (by avoiding flow CDC positive transplantation), the expanded indications for desensitization (patients who had negative DSA and flow CDC but with PRA ≥ 50% were given desensitization), and the lack of routine primary allograft nephrectomies (implying no massive reduction in immunosuppressive drugs after the return to dialysis) [15]. In addition, compared to other studies which mainly focused on third and fourth renal transplant patients, the majority of our retransplant population were second graft recipients, who may have fewer anti-HLA immunization and memory responses. Finally, the ethnic background of our patients differs from Caucasian and African American patients, which could influence transplant outcomes as well [16].

Notably, even with more potent Thymoglobulin induction therapy, the retransplant patients in our study had a much higher incidence of early acute TCMR compared to the first transplant group. This phenomenon has been rarely reported and emphasized in the literature. One study showed a relatively higher rate of both ABMR (22%) and TCMR (40%) in 37 presensitized kidney transplant patients without any desensitization treatment [17]. However, they mainly focused on ABMR in their study and did not discuss anything about the problem of acute TCMR. In the present study, we found that the type of acute TCMR had the following characteristics: (1) usually occurring around 2 weeks after the retransplantation; (2) repeated occurrence in some of the patients; and (3) the fact that it could be successfully reversed by high-dose steroids and/or Thymoglobulin. Since almost all these episodes of TCMR were effectively reversed, the short-term clinical outcome was not affected in any way. However, long-term graft survival still warrants further observation. The mechanisms for the elevated occurrence of early acute TCMR in retransplant patients also remain unclear. Memory T cells may play a major role in mediating the acute cellular rejection. In retransplant patients, there could be two potential approaches for the generation of alloreactive memory T cells: (1) direct alloantigenic stimulation of naïve T cells during the course of the previous transplantation and (2) homeostatic proliferation in response to transient lymphopenia resulting from T-cell depletion therapy, which induces the proliferation and differentiation of naïve T cells into memory cells [18]. In contrast to naïve T cells, memory T cells have lower activation thresholds and are less dependent on costimulation signals [19], are more resistant to killing by T-cell depletion therapy and regulation by regulatory T cells (Treg) [20], and are less susceptible to conventional immunosuppressive agents [21]. These features lead us to speculate that memory T cells may be responsible for the early acute TCMR observed in our retransplant patients. In the future, we will focus on memory T cells and try to provide direct evidence for our hypothesis. Additionally, we found that none of the highly presensitized retransplant patients with desensitization therapy developed early acute TCMR, indicating that desensitization may have some role in the prevention of early TCMR. However, we cannot draw a clear conclusion due to the small sample size in our current study.

In conclusion, our data on kidney retransplantation show excellent clinical outcomes with low incidence of early acute ABMR and satisfactory one-year patient and graft survival. However, the retransplant patients are at higher risk for the development of early acute TCMR, which requires accurate diagnosis and prompt treatment.

## Figures and Tables

**Table 1:** Demographics and early transplant outcomes.

	Second and subsequent transplant (*n* = 33), *n* (%)	First transplant (*n* = 90), *n* (%)	*P* value
Recipient age, year	46 ± 11	43 ± 11	0.170
Recipient gender			0.334
Male	25 (75.8)	60 (66.7)	
Female	8 (24.2)	30 (33.3)	
Type of donor			
Deceased	24 (72.7)	90 (100)	NA
Living-related	9 (27.3)	0	
Times of previous transplant			NA
1	28 (84.9)	0	
2	4 (12.1)	0	
3	1 (3.0)	0	
Cold ischemia time (h)	7.7 ± 5.8	8.6 ± 4.6	0.122
HLA-A, B, DR MM	3.3 ± 1.2	3.5 ± 0.8	0.261
Pretransplant PRA			<0.001
Class I and class II < 10%	14 (42.4)	87 (96.7)	
Class I or class II 10~50%	8 (24.3)	2 (2.2)	
Class I or class II ≥ 50%	11 (33.3)	1 (1.1)	
Preformed DSA+	3 (9.1)	0	NA
Delayed graft function	4 (12.1)	16 (17.8)	0.451
De novo DSA+	4 (12.1)	5 (5.6)	0.215
TCMR	10^*∗*^ (30.3)	5 (5.6)	<0.001
ABMR	2 (6.1)	4 (4.4)	0.712
1 yr graft survival	93.9%	92.2%	0.746
1 yr patient survival	100%	95.6%	0.218

^*∗*^4 of 10 patients had 2 episodes of acute TCMR.

**Table 2:** Impact of desensitization on incidence of TCMR in retransplant patients^#^.

	TCMR (+)	TCMR (−)	*P* value
	*n* (%)	*n* (%)	
PRA 10% < 50% (*n* = 21)	9 (42.9)	12 (57.1)	
PRA ≥ 50% (*n* = 10)	1^*∗*^ (10)	9 (90)	0.067
Nondesensitization (*n* = 24)	10 (41.7)	14 (58.3)	
Desensitization (*n* = 7)	0	7 (100)	<0.05

^#^Two patients with ABMR were censored from the analysis.

^*∗*^No desensitization due to HLA 0 MM.
